# Case 1/2016 - 56-Year-Old Male with Atrial Septal Defect, Pulmonary Arterial Hypertension, Hospitalized Due to Eisenmenger Syndrome

**DOI:** 10.5935/abc.20160035

**Published:** 2016-03

**Authors:** Carolina Santana, Antonio Augusto B. Lopes, Antonio Fernando Lins de Paiva, Luiz Alberto Benvenuti

**Affiliations:** Instituto do Coração (InCor) HC-FMUSP, São Paulo, SP - Brazil

**Keywords:** Heart Defects, Congenital, Heart Septal Defects, Atrial, Hypertension, Pulmonary, Eisenmenger Complex, Lung Diseases, Obstructive, Pulmonary Embolism

The patient is a 56-year-old male from the city of São Paulo, with atrial septal defect (ASD) and pulmonary arterial hypertension, who was hospitalized due to dyspnea, hypoxemia and lower limb edema.

In September 1996, the patient sought the Hospital do Coração for the first time due to fatigue on strenuous exertion for two months and chest X ray diagnosis of enlarged heart area.

His physical examination (September 11, 1996) revealed heart rate of 80 bpm and arterial blood pressure of 120/80 mm Hg. His pulmonary auscultation was normal. His cardiac auscultation showed a fixed and wide splitting of the second heart sound, and systolic heart murmur (+/4+) on the left sternal border.

He was diagnosed with ASD and pulmonary arterial hypertension.

The electrocardiogram (ECG - 1996) revealed right ventricular overload (Figure 1).

**Figure 1:**
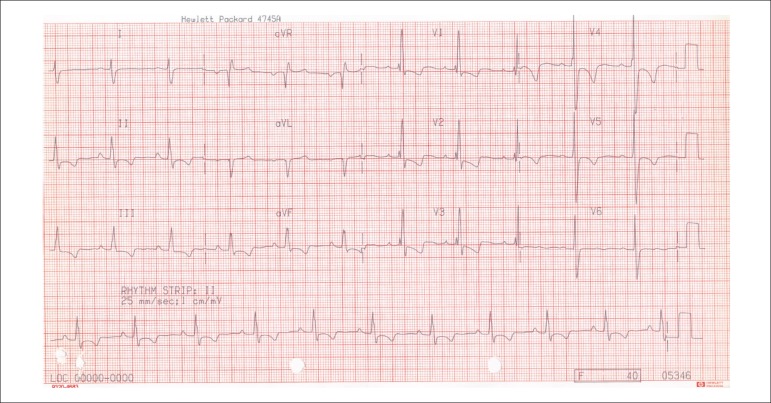
ECG (1996). Sinus rhythm, right ventricular overload.

The chest X ray showed bulging of the pulmonary artery, reduced pulmonary circulation and cardiomegaly (+/4+).

The echocardiogram (December 1996) revealed: septum and posterior wall thickness, 8 mm; left ventricular (LV) diameters (diastolic/systolic) of 50/30 mm; aortic, left atrial and right ventricular (RV) diameters of 35 mm, 40 mm and 54 mm, respectively; normal LV segmentary motility; RV hypokinesia; normal valves; and estimated pulmonary artery pressure of 84 mm Hg.

The pulmonary perfusion scan (1997) was interpreted as of low probability of pulmonary thromboembolism, and compatible with pulmonary hypertension.

The cardiac catheterization (July 22, 1997) revealed: ASD; pulmonary arterial hypertension; no obstructive coronary lesion; mild compression of the left main coronary artery by the pulmonary trunk; and LV hypertrophy with moderate diffuse hypokinesia. Table 1 shows the pressures and oximetry.

**Table 1:** Manometry and oximetry in cardiac catheterization

	**Manometry (mm Hg)**				**Oximetry (sat%)**
	**Syst**	** Diast 1 **	**Diast 2**	**Mean**	
RA				6	77.6
RV	80	0	5		76.1
PT	80	32		50	76.1
LA				5	93
LV	125	0	10		93
Ao	125	80		95	93.4

Syst: systole; Diast: diastole; RA: right atrium; RV: right ventricle; PT: pulmonary trunk; LA: left atrium; LV: left ventricle; Ao: aorta; Syst: systolic; Diast: diastolic.

The pulmonary hypertension was considered disproportional to the left-right shunt, and ASD correction was not indicated.

Warfarin, digoxin, furosemide and captopril were prescribed.

Other laboratory findings were as follows: hemoglobin, 19.3 g/dL; hematocrit, 56%; platelets, 176,000/mm^3^; and leukocytes, 8,000/mm^3^.

The patient remained stable with dyspnea on strenuous exertion until 2006, when he was 49 years old.

His laboratory tests in December 2005 showed: hemoglobin, 21.6 g/dL; hematocrit, 66%; platelets, 146,000/mm^3^; INR, 1.9; total cholesterol, 136 mg/dL; HDL, 45 mg/dL; LDL, 77 mg/dL; triglycerides, 68 mg/dL; glucose, 82 mg/dL; creatinine, 1 mg/dL; potassium, 4.4 mEq/L; sodium, 143 mEq/L; D dimer < 25 ng/mL.

The chest tomography showed signs of pulmonary emphysema, but no sign of pulmonary thromboembolism, suggesting pulmonary arterial hypertension.

The pulmonary function test (December 29, 2005) revealed: FVC= 2.77 L (94%); FEV_1_= 3.09 L (66%); FEF 25%-75%= 3.32 (32). After the bronchodilator: FVC= 3.81 L (101%); FEV_1_= 2.3 L (74%); FEF 25%-75%= 1.25 (38%). That result is compatible with mild obstruction (FEV1) and a marked reduction in the intermediate stage of breathing, FEF 25%-75%, compatible with obstruction of the small airways.

In June 2006, he experienced worsening of his dyspnea and chest pain, the diagnostic hypothesis of pulmonary thromboembolism being considered.

The pulmonary angiotomography (June 30, 2006) showed a filling gap in the lower lobe branch of the left pulmonary artery and another filling gap in the upper segmental branch to the left and parietal calcifications; mosaic perfusion area in the region; moderate pleural effusion on the left; and cardiomegaly. The measurements were as follows: pulmonary trunk, 47.9 mm; right pulmonary artery branch, 41 mm; left pulmonary artery branch, 36.2 mm.

One year after that episode, the patient sought medical care because of pain in the left hemithorax and cough.

The new tomography revealed a large thrombus in the pulmonary trunk, extending to the left and interlobar descending branch, eccentric, with marginal calcifications. In addition, the following were observed: smaller, marginal, distal thrombus in the right pulmonary artery; heterogeneous opacities and diffuse septal thickening in the left lung base; and pleural effusion to the left. The RV diameter/LV diameter ratio was close to 1. The measures were as follows: pulmonary trunk, 38.9 mm; right pulmonary artery, 42.4 mm; left pulmonary artery, 37.4 mm.

The patient was diagnosed with chronic pulmonary thromboembolism and pneumonia. Levofloxacin was prescribed.

The echocardiogram (July 12, 2007) showed marked biventricular systolic dysfunction, marked RV dilation and full-contrast filling of the cavity. No thrombus was visualized, and the RV outflow tract was dilated. Marked dilation of the pulmonary trunk and branches was identified, as was an echodense filamentous image in the pulmonary trunk suggestive of a thrombus. There was diffuse LV hypokinesia, and the emergence of the left coronary artery and its bifurcation were visualized, apparently with no sign of compression. In addition, marked dilation of the atria, moderate tricuspid regurgitation (could be underestimated due to RV dysfunction), moderate mitral regurgitation and bilateral pleural effusion were observed.

Two months later (September 2007), the patient sought the emergency of InCor with dyspnea at rest, tachycardia, hepatomegaly, ascitis and lower limb edema. The ECG showed atrial tachycardia. Congestive heart failure was diagnosed, requiring the use of intravenous dobutamine and furosemide, compensation being achieved. Spironolactone and amiodarone were added to the medications.

A new cardiac catheterization (September 2007) revealed: pulmonary artery pressure of 80/32 mm Hg (mean, 50 mm Hg); no coronary lesion; and diffuse, moderate-to-severe LV hypokinesia.

The patient was discharged with the prescription of furosemide (60 mg/day), spironolactone (25 mg/day), hydrochlorothiazide (25 mg/day), amiodarone (200 mg/day), warfarin (2.5 mg), clopidogrel (75 mg/day), omeprazol (40 mg/day) and fluid restriction of 1800 mL/day.

On the following month (October 2007), the patient was hospitalized again due to cardiac decompensation, when hypothyroidism was detected.

The new echocardiography (October 2007) showed right chamber dilation, ASD of 35 mm, moderate-to-severe tricuspid regurgitation, RV systolic pressure of 66 mm Hg, and dilation of the pulmonary arteries.

The abdominal ultrasonography (October 10, 2007) showed hepatomegaly with mild steatosis. Sildenafil (40 mg, 3x/day) was introduced, and the patient developed fatigue on strenuous exertion.

On physical examination (July 2011), the patient had a healthy coloring, was in a good general state of health, hydrated, cyanotic (3+/4+), anicteric, afebrile, eupneic. His room air saturation was 80%, heart rate, 76 bpm, arterial blood pressure, 120/80 mm Hg, and his chest anteroposterior diameter was enlarged. His pulmonary auscultation was normal. His cardiac auscultation revealed increased intensity of the second heart sound on the pulmonary area, and no heart murmur. The abdomen was normal, and there was no edema.

The ECG (2011) showed sinus rhythm with right ventricular overload (Figure 2).

**Figure 2:**
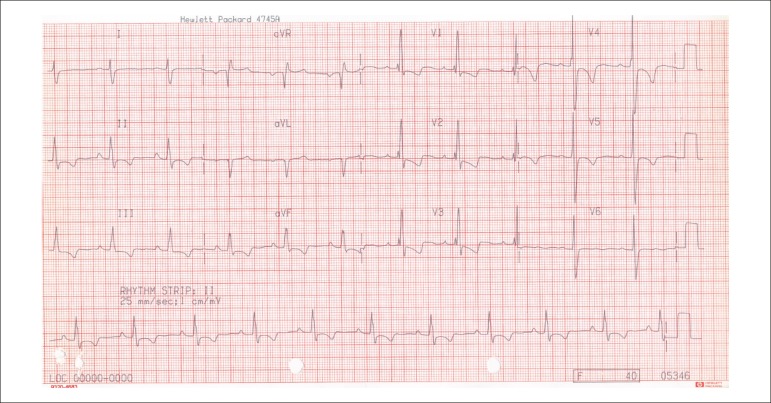
ECG (2011). Sinus rhythm, right ventricular overload.

The angiotomography of the pulmonary arteries (May 2011) revealed dilation of the pulmonary trunk (43 mm) and of the right and left pulmonary artery braches (37 mm and 36mm, respectively), suggesting pulmonary hypertension. The pulmonary trunk had parietal calcifications. There were circumferential contrast-filling defects, compatible with mural thrombi, in the left branch of the pulmonary trunk and ipsilateral interlobar artery. In addition, there was a filling defect associated with marked thinning of the left bronchial arteries of the upper lobe and of the arteries of the left basal segments, except for the artery of the posterior basal segment, findings compatible with previous thromboembolisms (Figures 3 and 4). (**Antonio Fernando Lins de Paiva, MD, radiologist**)

**Figure 3:**
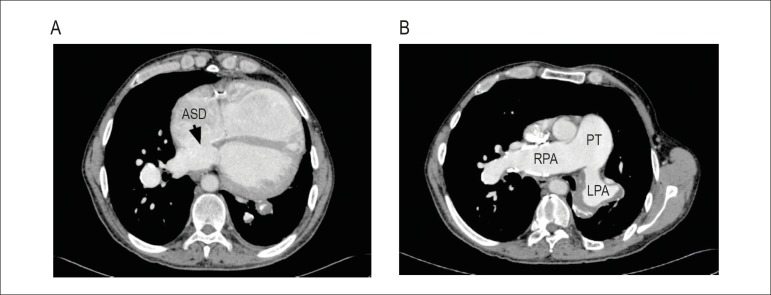
Angiotomography. A. Heart: right ventricular dilation and large atrial septal defect (ASD); B. Pulmonary arteries: dilated pulmonary trunk (PT), right pulmonary artery (RPA) and left pulmonary artery (LPA) with mural thrombi and calcifications.

**Figure 4:**
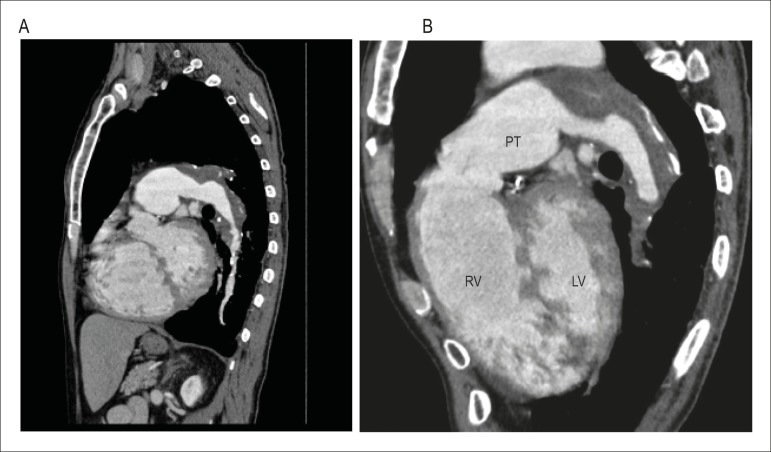
Chest angiotomography. A. Sagittal plane. Left pulmonary artery and left lower lobe branch - extensive mural thrombi and calcifications; B. Right ventricular outflow tract (RV) and dilated pulmonary trunk (PT); mural thrombi in the pulmonary artery.

The new echocardiogram (September 2011) was similar to that of 2007, except for the ASD size, which measured 24 mm. There was a mild reduction in the LV ejection fraction (LVEF= 52%).

Bosentan (125 mg, 2x/day) was added to the treatment, and the patient remained in functional class II until the end of 2012.

At the beginning of 2013, due to dyspnea worsening and an episode of pain in the left hemithorax, the patient sought help at a hospital close to his house, where he underwent bloodletting, and improved. His hematocrit was 67%.

The patient was admitted to the emergency unit on June 1, 2013, complaining of dyspnea worsening for 2 days, even on minimum exertion. He associated his symptoms with flu findings. He reported abdominal volume increase, lower limb edema, cyanosis worsening, reduced urine output, coughing up white mucus, and runny nose.

His examination showed: oxygen saturation blood test, 68%; heart rate, 85 bpm; arterial pressure, 99/60 mmHg; cyanosis, 3+/4+; diffusely reduced respiratory sounds with crepitant rales on the bases. The cardiac auscultation revealed reduced intensity of the rhythmic heart sounds, and fixed second heart sound splitting. The abdomen showed signs of ascitis and liver palpable 3 cm from the right costal border. There was lower limb edema (2+/4+). Dobutamine was administered (5 mcg/kg/min), being reduced to 3.3 mcg/kg/min on the following day, due to improved hemodynamic findings. Oseltamivir was used because of the previous history of flu.

The ECG showed right bundle-branch block and right ventricular overload (Figure 5).

**Figure 5:**
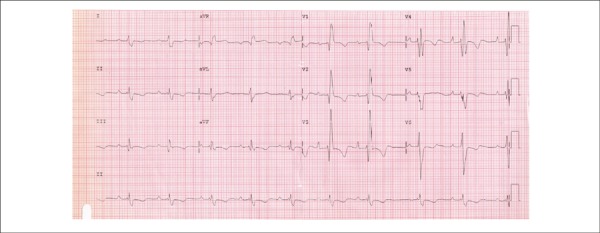
ECG (2012). Right bundle-branch block and right ventricular overload.

The chest X ray (June 01, 2013) showed an enlarged hilum, reduced peripheral lung vascularization, pulmonary bulging, heart enlargement (4+/4+) at the expense of the RV (Figure 6).

**Figure 6:**
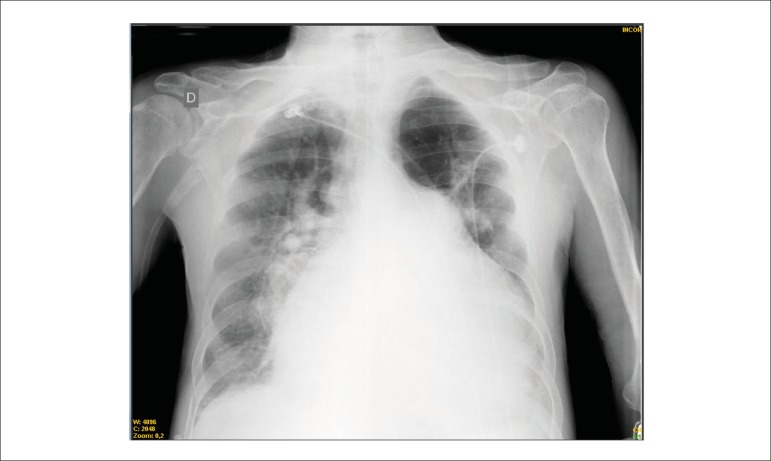
Chest X-ray (posteroanterior view) (June 01, 2013) - enlarged pulmonary hila, reduced peripheral pulmonary vascularization; cardiomegaly with enlargement of the left middle arch (enlarged pulmonary artery).

The laboratory tests (June 01, 2013) revealed: venous blood gas analysis: pH, 7.33; PCO_2_, 44.6 mm Hg; pO_2_, 29.1 mm Hg; oxygen saturation, 44.7%; bicarbonate, 22.8 mmol/L; base excess, (-) 3.3 mmol/L; sodium, 126 mEq/L; potassium, 3.8 mEq/L; lactate, 42 mg/dL; red blood cells, 6.9 million/mm^3^; hemoglobin, 22 g/dL; hematocrit, 63%; leukocytes, 5020/mm^3^ (5% band neutrophils, 60% segmented neutrophils, 29% lymphocytes, 6% monocytes); platelets, 424,000/mm^3^; creatinine, 0.92 mg/dL; urea, 34 mg/dL; magnesium, 1.40 mEq/L; C-reactive protein, 7.25 mg/L; TP (INR) 2.9; TTPA (rel) 1.49; D dimer, 536 ng/mL; fibrinogen, 186 mg/dL.

The echocardiogram (June 03, 2013) showed: RV dilation, 60 mm; left ventricle, 59x46 mm; moderately reduced LVEF (44%); ASD of 35 mm, with right-to-left flow; marked tricuspid regurgitation; RV systolic pressure, 50 mm Hg; marked mitral regurgitation; marked enlargement of the right atrium and moderate enlargement of the left atrium (at bedside, patient with oxygen catheter and on dobutamine) (Figure 7).

**Figure 7:**
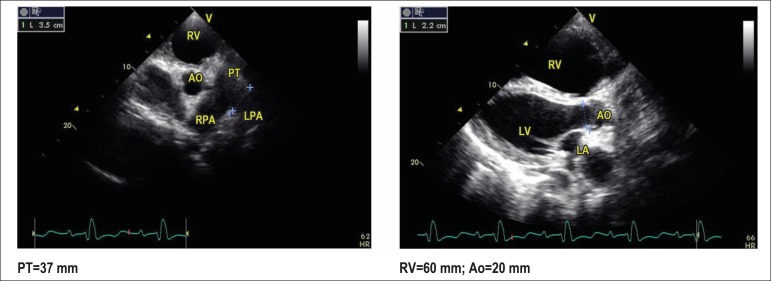
Transthoracic echocardiogram. Left: parasternal view in the short axis: dilation of pulmonary trunk (PT) and right and left branches of the pulmonary artery (RPA and LPA). Right: parasternal view in the long axis: right ventricle (RV) dilation and interventricular septal rectification.

On the sixth day of hospitalization (June 06, 2013), aggravation of the dyspnea, cyanosis and edema was observed, in addition to radiological worsening. Intravenous ceftriaxone, clarithromycin and furosemide were introduced, and dobutamine dose increased.

The laboratory tests (June 06, 2013) were as follows: urea, 20 mg/dL; creatinine, 0.83 mg/dL; sodium, 132 mEq/L; potassium, 4 mEq/L; venous lactate, 20 mg/dL; TP (INR) 2.6; TTPA (rel) 1.32; D dimer, 434 ng/mL; hemoglobin, 20,1 g/dL; hematocrit, 63%; leukocytes, 4460/mm^3^ (70% neutrophils, 1% eosinophils, 1% basophils, 15% lymphocytes and 13% monocytes); platelets, 117,000/mm^3^; albumin, 3.1 g/dL; C-reactive protein, 49.07 mg/L.

The chest X ray (June 06, 2013) showed opacification of the right lung and of the lower two-thirds of the left lung.

On the following night (June 07, 2013, 8PM), the patient had cardiac arrest with pulseless electrical activity. Cardiopulmonary resuscitation was initiated, and orotracheal intubation performed. On right hemithorax puncture, air was drained. The patient did not respond to the maneuvers and died.

## Clinical aspects

The ASD is the most common congenital heart disease in adulthood. It is characterized by a communication between the atria, being classified into four types (*ostium primum, ostium secundum, sinus venosus* and defect involving the coronary *sinus*). Only the *ostium secundum* defects are true ASD. The other types originate from defects in other structures, causing a communication between the atrial cavities.

The *ostium secundum* ASD is the most commonly found, corresponding to 80% of the cases, and results from the excessive resorption of the *septum primum* or deficient growth of the *septum secundum*.^1^

It usually has an insidious clinical presentation. The communication between the atria causes left-to-right shunt since birth; however, most children are asymptomatic. The malformation is detected on physical examination because of the presence of a systolic heart murmur on the left sternal border. Thus, the symptoms usually begin in adolescents or young adults. At the fifth decade of life, 75% to 80% of the patients are symptomatic.^1^

Our patient is a 56-year-old male diagnosed with ASD after the appearance of right heart failure symptoms. The diagnosis was late, when pulmonary hypertension secondary to that congenital malformation had already installed. At that stage, the patient already had reverse right-to-left shunt, and ASD correction was contraindicated.^1^

Surgical correction is indicated in the presence of a significant ASD or associated right ventricular overload, even when symptomless. The correction should be carefully assessed, because, when performed at a late stage, they can cause serious complications.^2^

When inadvertent, the ASD correction may worsen pulmonary hypertension, because the right-to-left shunt closure can increase pulmonary arterial pressure, aggravating the right heart failure. In addition, in more advanced cases, in which LV systolic dysfunction is associated, ASD closure increases pulmonary capillary pressure, causing pulmonary edema.^2^

Some parameters, such as normal oxygen saturation at rest or exertion, left-to-right shunt on echocardiography, and systolic pulmonary artery pressure lower than 70 mmHg, should be assessed before indicating ASD correction.^2^ None of these parameters were present in our patient.

To our patient, who had a late diagnosis of ASD complicated with pulmonary hypertension, surgical correction was contraindicated. The unrepaired ASD resulted in reverse right-to-left shunt, progressing to Eisenmenger syndrome, the final stage of pulmonary obstructive vascular disease secondary to the preexisting left-to-right shunt.^1^

The Eisenmenger syndrome is associated with several complications: arrhythmias, heart failure, pulmonary and systemic thrombosis, hemoptysis, renal dysfunction, cerebral abscess, gout and biliary lithiasis.^3^

At the time our patient was diagnosed, he already had right heart failure and RV dysfunction. As the disease progressed, episodes of pulmonary arterial thrombosis occurred. The prevalence of thrombosis is high in that population due to the hypoxemia that results in secondary polycythemia, and, consequently, higher risk for thrombosis. The chance of pulmonary arterial thrombosis is suggested to increase with age, probably due to the progression of endothelial dysfunction over time.^4^ Thus, to a patient at low risk for bleeding, anticoagulation should be discussed, even before thrombosis manifests.

The patient was medicated for heart failure, and oral anticoagulation was initiated even before the manifestation of pulmonary arterial thrombosis. He remained stable for 10 years, and, during which, his medication was optimized with the later introduction of pulmonary vasodilators. These drugs are essential to treat the Eisenmenger syndrome, improving functional class, increasing tolerance to exercise and improving performance on the 6-minute walking test.^5^

Despite adequate drug treatment, our patient's clinical status progressively worsened, and he died due to disease complications (heart failure and pulmonary arterial thrombosis).

This case shows the importance of the early diagnosis of ASD, allowing the repair of the congenital defect timely, before complications occur.

**(Carolina Santana, MD, and Prof. Antonio Augusto Lopes)**

**Diagnostic hypothesis:** end-stage pulmonary hypertension (Eisenmenger syndrome) due to atrial septal defect.

**(Carolina Santana, MD, and Prof. Antonio Augusto Lopes)**

## Postmortem examination

**Figure 8:**
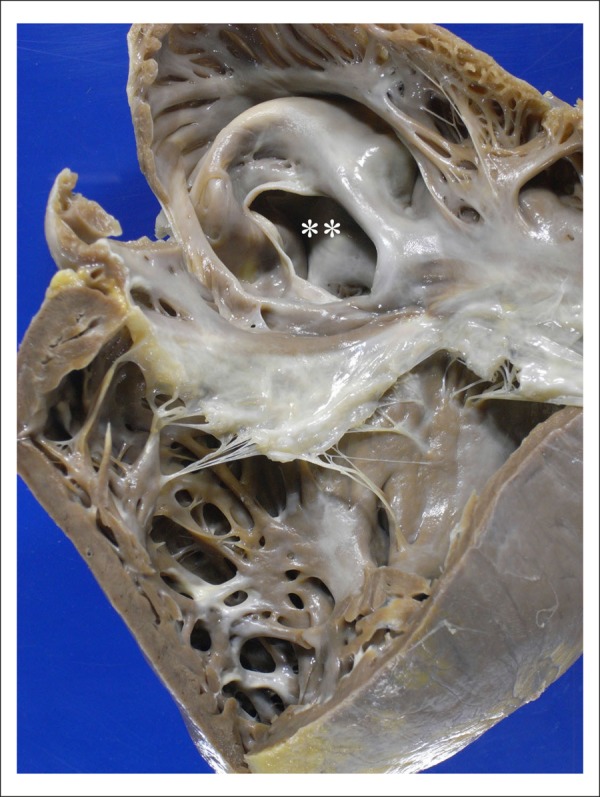
View of the right heart chambers opened, evidencing eccentric hypertrophy with marked dilation. Note the large atrial septal defect in the fossa ovalis (double asterisk).

**Figure 9:**
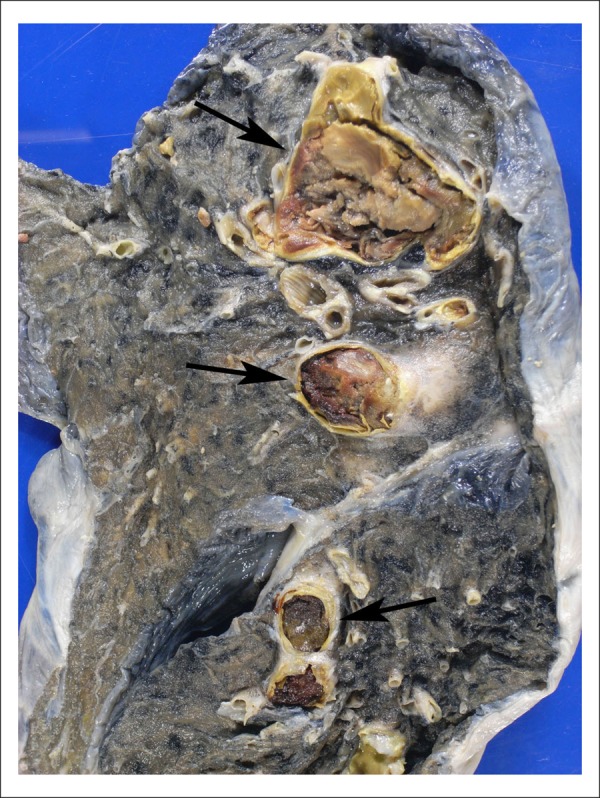
Gross examination. Section of the left lung showing atherosclerosis and calcification of the pulmonary artery branches, which are markedly dilated with extensive, occlusive, organized thrombosis (arrows).

**Figure 10:**
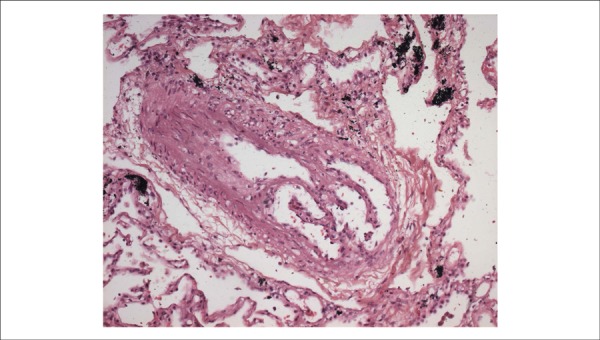
Microscopic section evidencing organized and recanalized arteriolar thrombosis of the lung parenchyma. Hematoxylin-Eosin, X 200.

**Figure 11:**
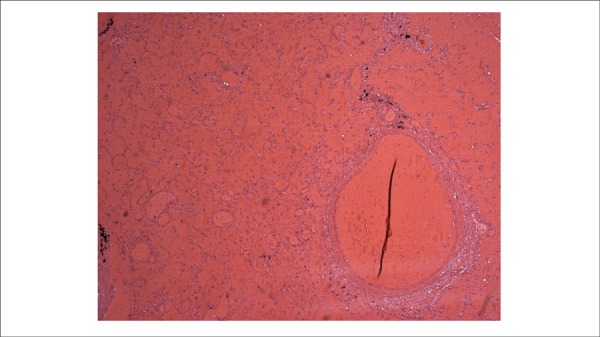
Microscopic section of the right lung lower lobe showing a recent hemorrhagic infarction. Hematoxylin-Eosin, X 50.

The heart was enlarged and globoid, weighing 832 g. On gross examination, the RV was prominent. The opening of the cavities evidenced eccentric hypertrophy of the four chambers, mainly the right ones, which showed marked dilation. A large ASD was identified in the *fossa ovalis* (*ostium secundum* type), measuring 3.5 x 3.0 cm (Figure 8). The ventricular septum was intact, no other heart malformation being identified. The microscopic examination revealed hypertrophy of the cardiomyocytes and myocardial interstitial fibrosis in both ventricles, with no evidence of ischemic lesions (infarction). The pulmonary trunk was dilated (diameter of 4.0 cm) and showed complicated atherosclerosis, with calcified areas. The right pulmonary artery showed calcified atherosclerosis, organized partial thrombosis, and markedly dilation of its parenchymatous branches. The process was similar in the left lung, but the thrombosis was more extensive, affecting numerous arterial branches, in an occlusive way, including the pulmonary hilum (Figure 9). The microscopic examination of the lungs confirmed the extensive organizing arterial thrombosis detected on gross examination, in addition to revealing: organized and rechanneled arteriolar thrombosis (Figure 10); recent focal thrombosis of a small artery and small hemorrhagic infarction in the lower lobe of the right lung (Figure 11); areas of alveolar hemorrhage; anthracosis; microscopic evidence of chronic bronchitis; and diffuse pulmonary emphysema. In addition, a small *in situ* adenocarcinoma was found in the right lung, measuring 9 mm. Moderate aorta atherosclerosis was identified, with calcified plaques. Neither pneumothorax nor bronchopneumonia were evidenced.

**(Luiz Alberto Benvenuti, MD)**

**Anatomopathological diagnoses** - Atrial septal defect in the *fossa ovalis*; secondary pulmonary hypertension; congestive heart failure with marked cardiomegaly; extensive organizing thrombosis in the pulmonary arteries and their branches; recent focal thrombosis of a small artery and small hemorrhagic infarction in the lower lobe of the right lung; pulmonary emphysema probably related to chronic smoking; *in situ* adenocarcinoma of the right lung.

**(Luiz Alberto Benvenuti, MD)**

## Comments

The patient was a 56-year-old male with wide ASD, marked secondary pulmonary hypertension and Eisenmenger syndrome. He was initially cared for at our service at the age of 37 years, when diagnosed with congenital heart defect. On that occasion, he already had marked pulmonary hypertension, and no indication for surgical repair. The surgical repair of congenital heart defects that progress with pulmonary hypertension should be early, before the latter acquires greater importance, becoming irreversible. Under certain circumstances, pulmonary biopsy is performed to grade hypertension, contributing to assess the possibility of surgical repair.^6,7^ Our patient progressed with congestive heart failure, polycythemia with marked hematocrit increase and episodes of thrombosis of pulmonary arteries. In addition, there was an inversion of the flow through the ASD (right-to-left), characterizing Einsenmenger syndrome.^8^

The postmortem examination confirmed the large ASD and pulmonary hypertension. There was marked dilation of the pulmonary arteries and branches, with extensive organized thrombosis, affecting the hilar vessels. These findings can occur in pulmonary hypertension of any etiology, but, in our case, the thrombosis was exuberant, probably related to the marked increase in the patient's hematocrit and prothrombotic state, described in the adult's Eisenmenger syndrome.^8,9^ It is worth noting that, under those circumstances, there is thrombosis rather than thromboembolism of the pulmonary arteries, because it is a local phenomenon and not embolization of thrombi formed in another vascular territory. Death resulted from cardiogenic shock, due to heart failure and extensive thrombosis of the pulmonary arteries, aggravated by hemorrhagic pulmonary infarction.

**(Luiz Alberto Benvenuti, MD)**

**Editor da Seção:** Alfredo José Mansur (ajmansur@incor.usp.br)

**Editores Associados:** Desidério Favarato (dclfavarato@incor.usp.br)

Vera Demarchi Aiello (anpvera@incor.usp.br)
